# Erratum: Group Testing for SARS-CoV-2 Allows for Up to 10-Fold Efficiency Increase Across Realistic Scenarios and Testing Strategies

**DOI:** 10.3389/fpubh.2021.781326

**Published:** 2021-10-18

**Authors:** Frontiers Production Office

**Affiliations:** Frontiers Media SA, Lausanne, Switzerland

**Keywords:** group testing, SARS-CoV-2, pooling, COVID-19, informative testing, RT-PCR

Due to a production error, the wrong HTML link was provided in the article abstract.

Instead of “www.grouptexting.com,” the link should be “www.group-testing.com.”

The publisher apologizes for this mistake. The original article has been updated.

