# Supplementary material for: Group Testing for SARS-CoV-2 Allows for Up to 10-Fold Efficiency Increase Across Realistic Scenarios and Testing Strategies
Source: Front Public Health. 2021 Aug 18;9:583377. doi: 10.3389/fpubh.2021.583377 (PMC8416485; doi:10.3389/fpubh.2021.583377)
Supplement: Supplementary file 1 [file Data_Sheet_1.pdf]

APPENDIX

Table 1. Illustration of the expected total amount of tests needed for testing in three different cities by assuming specificity and sensitivity of 99%. All numbers are in million.

	Munich (population: 1.47)				
p	D2	D3	A2	$A1_2$	$A1_3$
0.025%	0.112	0.097	0.207	0.184	0.276
0.05%	0.118	0.101	0.207	0.184	0.276
0.075%	0.123	0.104	0.206	0.184	0.276
0.1%	0.129	0.107	0.206	0.184	0.276
0.2%	0.151	0.119	0.204	0.184	0.276
0.3%	0.173	0.131	0.204	0.184	0.276
0.4%	0.196	0.141	0.206	0.184	0.276
0.5%	0.218	0.153	0.209	0.184	0.276
1.0%	0.298	0.207	0.232	0.184	0.276
1.5%	0.362	0.260	0.269	0.184	0.276
2.0%	0.413	0.310	0.315	0.226	0.276
2.5%	0.459	0.357	0.363	0.294	0.276
3.0%	0.500	0.404	0.407	0.326	0.294
	Vienna (population: 1.90)				
p	D2	D3	A2	$A1_2$	$A1_3$
0.025%	0.144	0.125	0.268	0.238	0.357
0.05%	0.152	0.131	0.268	0.238	0.357
0.075%	0.160	0.135	0.266	0.238	0.357
0.1%	0.167	0.139	0.266	0.238	0.357
0.2%	0.196	0.154	0.264	0.238	0.357
0.3%	0.224	0.169	0.264	0.238	0.357
0.4%	0.253	0.182	0.266	0.238	0.357
0.5%	0.281	0.198	0.270	0.238	0.357
1.0%	0.386	0.268	0.300	0.238	0.357
1.5%	0.467	0.336	0.348	0.238	0.357
2.0%	0.534	0.401	0.407	0.293	0.357
2.5%	0.593	0.462	0.469	0.380	0.357
3.0%	0.646	0.522	0.526	0.422	0.380
	Rio de Janeiro (population: 6.69)				
p	D2	D3	A2	$A1_2$	$A1_3$
0.025%	0.508	0.442	0.943	0.836	1.258
0.05%	0.535	0.462	0.943	0.836	1.258
0.075%	0.562	0.475	0.937	0.836	1.258
0.1%	0.589	0.488	0.937	0.836	1.258
0.2%	0.689	0.542	0.930	0.836	1.258
0.3%	0.789	0.595	0.930	0.836	1.258
0.4%	0.890	0.642	0.937	0.836	1.258
0.5%	0.990	0.696	0.950	0.836	1.258
1.0%	1.358	0.943	1.057	0.836	1.258
1.5%	1.646	1.184	1.224	0.836	1.258
2.0%	1.880	1.412	1.432	1.030	1.258
2.5%	2.087	1.626	1.652	1.338	1.258
3.0%	2.275	1.840	1.853	1.485	1.338

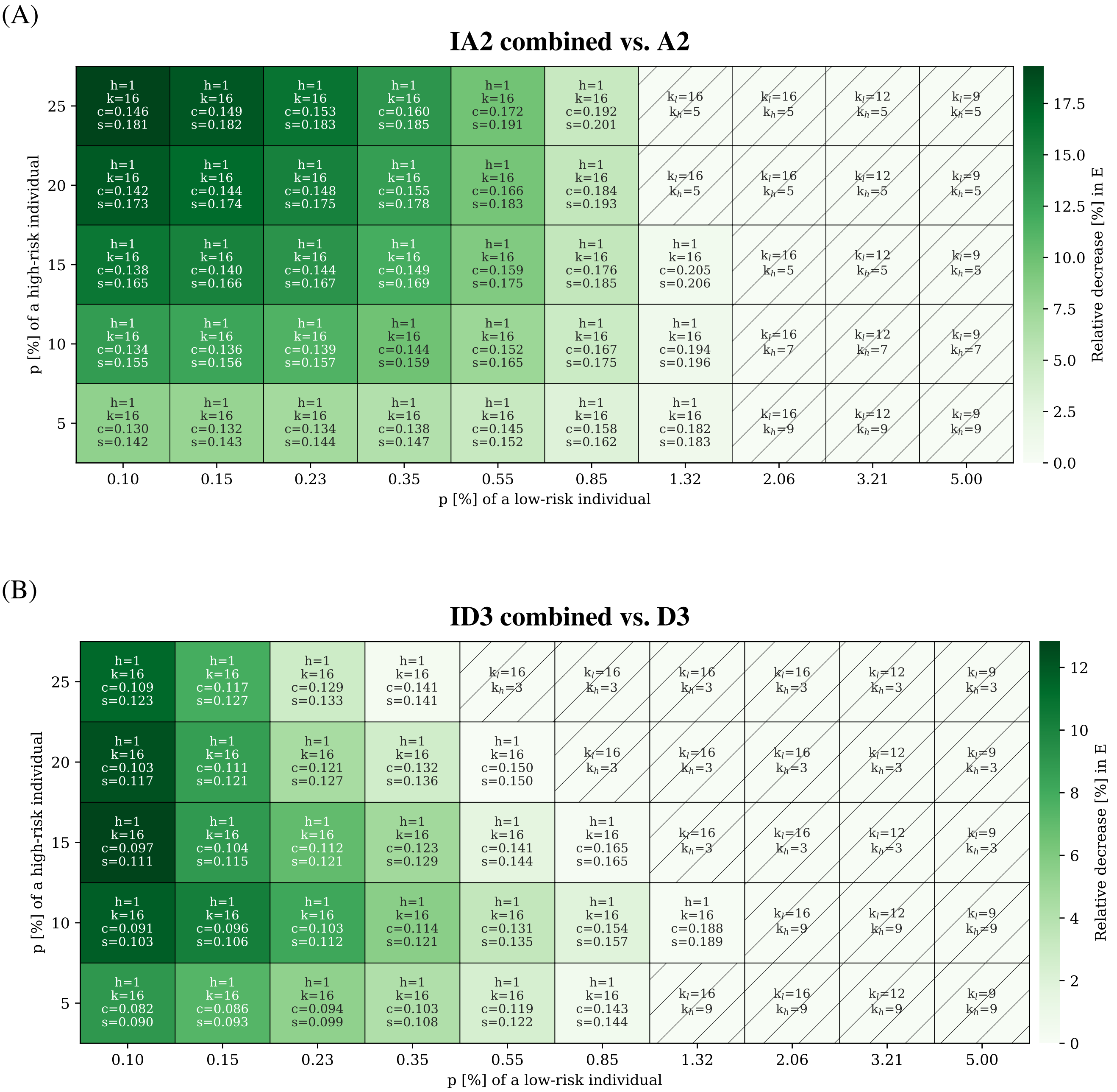

Figure A 1. Comparison between different informative methods when the maximum group size is assumed to be 16. The dark green tiles indicate scenarios where pooling one individual of a high-risk group with specimens from low-risk groups reduces the average amount of tests needed over testing them separately. h represents the number of high-risk individuals per group, c/s is expected number of tests per person for combined/separate testing respectively and $k/k_l/k_h$ are the optimal group sizes for combined/separate-low/separate-high testing. The hatched tiles indicate when this strategy should not be adopted. The PCR test is assumed to have $S_e = S_p = 100\%$.

Table 2. Overview of the optimal group size (for each stage i) for different hierarchical methods and infection rates p. Besides E, the expected number of tests per person, $TIpT$ gives the average amount of tested individuals per test. $S_e(.)$/$S_p(.)$ denotes, respectively, the sensitivity/specificity of the method for optimal group size. (Assumption: $S_p = 0.99$, **left** table: $S_e = 0.99$, **right** table $S_e = 0.90$)

Method	p	k_1	k_2	k_3	E	$TIpT$	$S_e(.)$	$S_p(.)$
D2	0.025%	16	1	0	0.076	13.09	98%	100%
	0.05%	16	1	0	0.08	12.45	98%	100%
	0.075%	16	1	0	0.084	11.88	98%	100%
	0.1%	16	1	0	0.088	11.35	98%	100%
	0.2%	16	1	0	0.103	9.67	98%	100%
	0.3%	16	1	0	0.118	8.44	98%	100%
	0.4%	16	1	0	0.133	7.50	98%	99.9%
	0.5%	15	1	0	0.148	6.78	98%	99.9%
	1%	11	1	0	0.204	4.91	98%	99.9%
	1.5%	9	1	0	0.246	4.07	98%	99.9%
	2%	8	1	0	0.281	3.55	98%	99.9%
	2.5%	7	1	0	0.312	3.21	98%	99.9%
	3%	6	1	0	0.34	2.94	98%	99.9%
	4%	6	1	0	0.37	2.70	81%	99.8%
	5%	5	1	0	0.411	2.43	81%	99.8%
	6%	5	1	0	0.447	2.24	81%	99.8%
	7%	5	1	0	0.481	2.08	81%	99.8%
	8%	4	1	0	0.512	1.95	81%	99.8%
	9%	4	1	0	0.54	1.85	81%	99.8%
	10%	4	1	0	0.566	1.77	81%	99.8%
	11%	4	1	0	0.592	1.69	81%	99.7%
	12%	4	1	0	0.616	1.62	81%	99.7%
	13%	4	1	0	0.64	1.56	81%	99.7%
	14%	4	1	0	0.663	1.51	81%	99.7%
	15%	4	1	0	0.685	1.46	81%	99.7%
D3	0.025%	16	8	1	0.066	15.08	97%	100%
	0.05%	16	6	1	0.069	14.58	97%	100%
	0.075%	16	6	1	0.071	14.14	97%	100%
	0.1%	16	6	1	0.073	13.74	97%	100%
	0.2%	16	4	1	0.081	12.36	97%	100%
	0.3%	16	4	1	0.089	11.29	97%	100%
	0.4%	16	4	1	0.096	10.37	97%	100%
	0.5%	16	4	1	0.104	9.62	97%	100%
	1%	16	4	1	0.141	7.08	97%	100%
	1.5%	16	4	1	0.177	5.66	97%	100%
	2%	16	4	1	0.211	4.74	97%	99.9%
	2.5%	16	4	1	0.243	4.11	97%	99.9%
	3%	12	3	1	0.274	3.64	97%	99.9%
	4%	12	3	1	0.329	3.04	97%	99.9%
	5%	9	3	1	0.377	2.65	97%	99.9%
	6%	9	3	1	0.423	2.37	97%	99.9%
	7%	9	3	1	0.466	2.15	97%	99.9%
	8%	9	3	1	0.507	1.97	97%	99.9%
	9%	9	3	1	0.546	1.83	97%	99.8%
	10%	9	3	1	0.584	1.71	97%	99.8%
	11%	9	3	1	0.619	1.61	97%	99.8%
	12%	4	1	1	0.652	1.53	98%	99.7%
	13%	3	1	1	0.678	1.47	98%	99.8%
	14%	3	1	1	0.7	1.43	98%	99.7%
	15%	3	1	1	0.722	1.39	98 %	99.7%

Method	p	k_1	k_2	k_3	E	$TIpT$	$S_e(.)$	$S_p(.)$
D2	0.025%	16	1	0	0.076	13.14	81%	100%
	0.05%	16	1	0	0.08	12.56	81%	100%
	0.075%	16	1	0	0.083	12.03	81%	100%
	0.1%	16	1	0	0.087	11.55	81%	100%
	0.2%	16	1	0	0.101	9.94	81%	100%
	0.3%	16	1	0	0.114	8.75	81%	100%
	0.4%	16	1	0	0.128	7.82	81%	99.9%
	0.5%	16	1	0	0.141	7.09	81%	99.9%
	1%	11	1	0	0.194	5.15	81%	99.9%
	1.5%	9	1	0	0.234	4.27	81%	99.9%
	2%	8	1	0	0.268	3.73	81%	99.9%
	2.5%	7	1	0	0.297	3.36	81%	99.9%
	3%	7	1	0	0.324	3.09	81%	99.8%
	4%	6	1	0	0.37	2.70	81%	99.8%
	5%	5	1	0	0.411	2.43	81%	99.8%
	6%	5	1	0	0.447	2.24	81%	99.8%
	7%	5	1	0	0.481	2.08	81%	99.8%
	8%	4	1	0	0.512	1.95	81%	99.8%
	9%	4	1	0	0.54	1.85	81%	99.8%
	10%	4	1	0	0.566	1.77	81%	99.8%
	11%	4	1	0	0.592	1.69	81%	99.7%
	12%	4	1	0	0.616	1.62	81%	99.7%
	13%	4	1	0	0.64	1.56	81%	99.7%
	14%	4	1	0	0.663	1.51	81%	99.7%
	15%	4	1	0	0.685	1.46	81%	99.7%
D3	0.025%	16	8	1	0.066	15.17	72.9%	100%
	0.05%	16	8	1	0.068	14.71	72.9%	100%
	0.075%	16	6	1	0.07	14.33	72.9%	100%
	0.1%	16	6	1	0.072	13.97	72.9%	100%
	0.2%	16	6	1	0.079	12.72	72.9%	100%
	0.3%	16	4	1	0.086	11.70	72.9%	100%
	0.4%	16	4	1	0.092	10.85	72.9%	100%
	0.5%	16	4	1	0.099	10.12	72.9%	100%
	1%	16	4	1	0.131	7.63	72.9%	100%
	1.5%	16	4	1	0.162	6.18	72.9%	100%
	2%	16	4	1	0.191	5.23	72.9%	100%
	2.5%	16	4	1	0.219	4.56	72.9%	99.9%
	3%	16	4	1	0.246	4.06	72.9%	99.9%
	4%	12	4	1	0.296	3.38	72.9%	99.9%
	5%	12	4	1	0.341	2.93	72.9%	99.9%
	6%	9	3	1	0.381	2.63	72.9%	99.9%
	7%	9	3	1	0.418	2.39	72.9%	99.9%
	8%	9	3	1	0.453	2.21	72.9%	99.9%
	9%	9	3	1	0.487	2.05	72.9%	99.9%
	10%	9	3	1	0.519	1.93	72.9%	99.8%
	11%	9	3	1	0.549	1.82	72.9%	99.8%
	12%	9	3	1	0.578	1.73	72.9%	99.8%
	13%	9	3	1	0.606	1.65	72.9%	99.8%
	14%	9	3	1	0.633	1.58	72.9%	99.8%
	15%	12	3	1	0.658	1.52	72.9%	99.8%

Table 3. Overview of the optimal group size (for each stage i) for array testing and infection rates p. Besides E, the expected number of tests per person, $TIpT$ gives the average amount of tested individuals per test. $S_e(.)/S_p(.)$ denotes, respectively, the sensitivity/specificity of the method for optimal group size[15]. (Assumption: $S_p = 0.99$, **left** table: $S_e = 0.99$, **right** table $S_e = 0.90$)

Method	p	k_1	k_2	k_3	E	$TIpT$	$S_e(.)$	$S_p(.)$
A2	0.025%	16	1	0	0.142	7.07	98.6%	100%
	0.05%	16	1	0	0.141	7.10	98.5%	100%
	0.075%	16	1	0	0.14	7.12	98.4%	100%
	0.1%	16	1	0	0.14	7.14	98.4%	100%
	0.2%	16	1	0	0.139	7.19	98.1%	100%
	0.3%	16	1	0	0.139	7.19	97.9%	100%
	0.4%	16	1	0	0.14	7.14	97.7%	100%
	0.5%	16	1	0	0.142	7.06	97.5%	100%
	1%	16	1	0	0.158	6.34	97.2%	100%
	1.5%	16	1	0	0.183	5.46	97.1%	100%
	2%	16	1	0	0.214	4.67	97%	99.9%
	2.5%	14	1	0	0.247	4.05	97%	99.9%
	3%	13	1	0	0.277	3.61	97%	99.9%
	4%	11	1	0	0.331	3.02	97%	99.9%
	5%	10	1	0	0.381	2.63	97%	99.9%
	6%	9	1	0	0.426	2.35	97%	99.9%
	7%	8	1	0	0.468	2.14	97.1%	99.8%
	8%	7	1	0	0.509	1.97	97.1%	99.8%
	9%	7	1	0	0.545	1.83	97.1%	99.8%
	10%	7	1	0	0.582	1.72	97%	99.8%
	11%	6	1	0	0.616	1.62	97.1%	99.8%
	12%	6	1	0	0.648	1.54	97.1%	99.8%
	13%	6	1	0	0.68	1.47	97.1%	99.8%
	14%	6	1	0	0.712	1.41	97%	99.7%
	15%	5	1	0	0.742	1.35	97.1%	99.8%

Method	p	k_1	k_2	k_3	E	$TIpT$	$S_e(.)$	$S_p(.)$
A2	0.025%	16	1	0	0.142	7.04	86.1%	100%
	0.05%	16	1	0	0.142	7.04	85.4%	100%
	0.075%	16	1	0	0.142	7.05	84.8%	100%
	0.1%	16	1	0	0.142	7.05	84.1%	100%
	0.2%	16	1	0	0.142	7.05	82%	100%
	0.3%	16	1	0	0.142	7.03	80.2%	100%
	0.4%	16	1	0	0.143	6.99	78.8%	100%
	0.5%	16	1	0	0.144	6.94	77.6%	100%
	1%	16	1	0	0.156	6.43	74.5%	100%
	1.5%	16	1	0	0.175	5.72	73.5%	100%
	2%	16	1	0	0.2	5.01	73.1%	99.9%
	2.5%	16	1	0	0.228	4.38	73%	99.9%
	3%	14	1	0	0.256	3.91	73%	99.9%
	4%	12	1	0	0.306	3.27	73%	99.9%
	5%	11	1	0	0.351	2.85	73%	99.9%
	6%	10	1	0	0.392	2.55	73%	99.9%
	7%	9	1	0	0.43	2.32	73.1%	99.8%
	8%	8	1	0	0.467	2.14	73.2%	99.8%
	9%	8	1	0	0.5	2.00	73.1%	99.8%
	10%	7	1	0	0.534	1.87	73.2%	99.8%
	11%	7	1	0	0.563	1.78	73.1%	99.8%
	12%	7	1	0	0.593	1.69	73.1%	99.8%
	13%	7	1	0	0.622	1.61	73%	99.7%
	14%	6	1	0	0.649	1.54	73.2%	99.8%
	15%	6	1	0	0.674	1.48	73.2%	99.7%

[15] One could expect that the expected number of tests per person of a group testing method does not improve for an increasing prevalence. Nevertheless, Table 3 indicates the opposite for low infection rates and sensitivity $S_e = 0.99$. While for 0.1% prevalence 0.140 tests per individual are expected, this improves to $E = 0.139$ for $p = 0.2\%$. The explanation for such oscillatory pattern comes from the general implementation of A2's expected number of tests per person in the binGroup package where plausibility checks are done. As mentioned in Subsection 3.2.3 for $S_e, S_p < 1$, in a scenario where a positive row/column group but not a single positive column/row group are found, individual tests of the positive row/column group should be performed. In a low prevalence setting, those additional tests have a higher impact and lead to the oscillation. As a side note, from a theoretical perspective, the oscillations in the expected number of tests per person for low prevalence do not contradict the theorem by Yao and Hwang (A fundamental monotonicity in group testing. SIAM J Discrete Math. 1988; 1(2):256–259. `https://doi.org/10.1137/0401026`) since the theorem concerns the minimum over all possible strategies and this one, even though can be a very good method for the current purposes, does not achieve the theoretical minimum.

Table 4. Overview of the optimal group size (for each stage i) for different non-adaptive methods and infection rates p. Besides E, the expected number of tests per person, $TIpT$ gives the average amount of tested individuals per test. $S_e(.)$/$S_p(.)$ denotes, respectively, the sensitivity/specificity of the method for optimal group size. (Assumption: $S_p = 0.99$, **left** table: $S_e = 0.99$, **right** table $S_e = 0.90$)

Method	p	k_1	k_2	k_3	E	$TIpT$	$S_e(.)$	$S_p(.)$
$A1_2$	0.025%	16	0	0	0.125	8.00	98%	100%
	0.05%	16	0	0	0.125	8.00	98%	100%
	0.075%	16	0	0	0.125	8.00	98%	100%
	0.1%	16	0	0	0.125	8.00	98%	99.9%
	0.2%	16	0	0	0.125	8.00	98%	99.9%
	0.3%	16	0	0	0.125	8.00	98%	99.7%
	0.4%	16	0	0	0.125	8.00	98%	99.6%
	0.5%	16	0	0	0.125	8.00	98%	99.3%
	1%	16	0	0	0.125	8.00	98%	97.8%
	1.5%	16	0	0	0.125	8.00	98%	95.6%
	2%	13	0	0	0.154	6.50	98%	95.1%
	2.5%	10	0	0	0.2	5.00	98%	95.6%
	3%	9	0	0	0.222	4.50	98%	95.1%
	4%	7	0	0	0.286	3.50	98%	95%
	5%	5	0	0	0.4	2.50	98%	96.3%
	6%	4	0	0	0.5	2.00	98%	96.9%
	7%	4	0	0	0.5	2.00	98%	95.9%
	8%	3	0	0	0.667	1.50	98%	97.4%
	9%	3	0	0	0.667	1.50	98%	96.8%
	10%	3	0	0	0.667	1.50	98%	96.2%
	11%	3	0	0	0.667	1.50	98%	95.4%
	12%	2	0	0	1	1.00	98%	98.4%
	13%	2	0	0	1	1.00	98%	98.1%
	14%	2	0	0	1	1.00	98%	97.8%
	15%	2	0	0	1	1.00	98%	97.5%
$A1_3$	0.025%	16	0	0	0.188	5.33	97%	100%
	0.05%	16	0	0	0.188	5.33	97%	100%
	0.075%	16	0	0	0.188	5.33	97%	100%
	0.1%	16	0	0	0.188	5.33	97%	100%
	0.2%	16	0	0	0.188	5.33	97%	100%
	0.3%	16	0	0	0.188	5.33	97%	100%
	0.4%	16	0	0	0.188	5.33	97%	100%
	0.5%	16	0	0	0.188	5.33	97%	100%
	1%	16	0	0	0.188	5.33	97%	99.7%
	1.5%	16	0	0	0.188	5.33	97%	99.1%
	2%	16	0	0	0.188	5.33	97%	98.1%
	2.5%	16	0	0	0.188	5.33	97%	96.7%
	3%	15	0	0	0.2	5.00	97%	95.7%
	4%	12	0	0	0.25	4.00	97%	95.2%
	5%	9	0	0	0.333	3.00	97%	96.1%
	6%	8	0	0	0.375	2.67	97%	95.5%
	7%	7	0	0	0.429	2.33	97%	95.5%
	8%	6	0	0	0.5	2.00	97%	95.9%
	9%	5	0	0	0.6	1.67	97%	96.8%
	10%	5	0	0	0.6	1.67	97%	95.8%
	11%	4	0	0	0.75	1.33	97%	97.3%
	12%	4	0	0	0.75	1.33	97%	96.7%
	13%	4	0	0	0.75	1.33	97%	95.9%
	14%	4	0	0	0.75	1.33	97%	95.1%
	15%	3	0	0	1	1.00	97%	97.8%

Method	p	k_1	k_2	k_3	E	$TIpT$	$S_e(.)$	$S_p(.)$
$A1_2$	0.025%	16	0	0	0.125	8.00	81%	100%
	0.05%	16	0	0	0.125	8.00	81%	100%
	0.075%	16	0	0	0.125	8.00	81%	100%
	0.1%	16	0	0	0.125	8.00	81%	100%
	0.2%	16	0	0	0.125	8.00	81%	99.9%
	0.3%	16	0	0	0.125	8.00	81%	99.8%
	0.4%	16	0	0	0.125	8.00	81%	99.6%
	0.5%	16	0	0	0.125	8.00	81%	99.5%
	1%	16	0	0	0.125	8.00	81%	98.2%
	1.5%	16	0	0	0.125	8.00	81%	96.4%
	2%	14	0	0	0.143	7.00	81%	95.3%
	2.5%	11	0	0	0.182	5.50	81%	95.6%
	3%	10	0	0	0.2	5.00	81%	95%
	4%	7	0	0	0.286	3.50	81%	95.9%
	5%	6	0	0	0.333	3.00	81%	95.5%
	6%	5	0	0	0.4	2.50	81%	95.8%
	7%	4	0	0	0.5	2.00	81%	96.6%
	8%	4	0	0	0.5	2.00	81%	95.7%
	9%	3	0	0	0.667	1.50	81%	97.3%
	10%	3	0	0	0.667	1.50	81%	96.8%
	11%	3	0	0	0.667	1.50	81%	96.2%
	12%	3	0	0	0.667	1.50	81%	95.6%
	13%	2	0	0	1	1.00	81%	98.4%
	14%	2	0	0	1	1.00	81%	98.2%
	15%	2	0	0	1	1.00	81%	97.9%
$A1_3$	0.025%	16	0	0	0.188	5.33	72.9%	100%
	0.05%	16	0	0	0.188	5.33	72.9%	100%
	0.075%	16	0	0	0.188	5.33	72.9%	100%
	0.1%	16	0	0	0.188	5.33	72.9%	100%
	0.2%	16	0	0	0.188	5.33	72.9%	100%
	0.3%	16	0	0	0.188	5.33	72.9%	100%
	0.4%	16	0	0	0.188	5.33	72.9%	100%
	0.5%	16	0	0	0.188	5.33	72.9%	100%
	1%	16	0	0	0.188	5.33	72.9%	99.8%
	1.5%	16	0	0	0.188	5.33	72.9%	99.3%
	2%	16	0	0	0.188	5.33	72.9%	98.6%
	2.5%	16	0	0	0.188	5.33	72.9%	97.5%
	3%	16	0	0	0.188	5.33	72.9%	96.2%
	4%	13	0	0	0.231	4.33	72.9%	95.5%
	5%	11	0	0	0.273	3.67	72.9%	95%
	6%	9	0	0	0.333	3.00	72.9%	95.4%
	7%	8	0	0	0.375	2.67	72.9%	95.2%
	8%	7	0	0	0.429	2.33	72.9%	95.3%
	9%	6	0	0	0.5	2.00	72.9%	95.9%
	10%	5	0	0	0.6	1.67	72.9%	96.8%
	11%	5	0	0	0.6	1.67	72.9%	96%
	12%	5	0	0	0.6	1.67	72.9%	95.1%
	13%	4	0	0	0.75	1.33	72.9%	96.9%
	14%	4	0	0	0.75	1.33	72.9%	96.3%
	15%	4	0	0	0.75	1.33	72.9%	95.6%

Table 5. Overview of the sensitivity $S_e(.)$ and specificity $S_p(.)$ for different S_e of qRT-PCR with a fixed prevalence assumption of $p = 3\%$ and specificity $S_p = 0.99\%$.

Method	S_e	$S_e(.)$	$S_p(.)$	k_1
D2	100%	100%	99.9%	6
	99%	98%	99.9%	6
	98%	96%	99.9%	6
	97%	94.1%	99.9%	6
	96%	92.2%	99.8%	7
	95%	90.2%	99.8%	7
	94%	88.4%	99.8%	7
	93%	86.5%	99.8%	7
	92%	84.6%	99.8%	7
	91%	82.8%	99.8%	7
	90%	81%	99.8%	7
D3	100%	100%	99.9%	12
	99%	97%	99.9%	12
	98%	94.1%	99.9%	16
	97%	91.3%	99.9%	16
	96%	88.5%	99.9%	16
	95%	85.7%	99.9%	16
	94%	83.1%	99.9%	16
	93%	80.4%	99.9%	16
	92%	77.9%	99.9%	16
	91%	75.4%	99.9%	16
	90%	72.9%	99.9%	16
A2	100%	100%	99.9%	13
	99%	97%	99.9%	13
	98%	94.2%	99.9%	13
	97%	91.3%	99.9%	13
	96%	88.5%	99.9%	13
	95%	85.8%	99.9%	13
	94%	83.2%	99.9%	13
	93%	80.5%	99.9%	14
	92%	77.9%	99.9%	14
	91%	75.5%	99.9%	14
	90%	73%	99.9%	14

Method	S_e	$S_e(.)$	$S_p(.)$	k_1
$A1_2$	100%	100%	96%	8
	99%	98%	95.1%	9
	98%	96%	95.2%	9
	97%	94.1%	95.3%	9
	96%	92.2%	95.4%	9
	95%	90.2%	95.5%	9
	94%	88.4%	95.5%	9
	93%	86.5%	95.6%	9
	92%	84.6%	95.7%	9
	91%	82.8%	95.8%	9
	90%	81%	95%	10
$A1_3$	100%	100%	95.6%	15
	99%	97%	95.7%	15
	98%	94.1%	95.1%	16
	97%	91.3%	95.3%	16
	96%	88.5%	95.4%	16
	95%	85.7%	95.5%	16
	94%	83.1%	95.7%	16
	93%	80.4%	95.8%	16
	92%	77.9%	95.9%	16
	91%	75.4%	96.1%	16
	90%	72.9%	96.2%	16
