# Supplementary figures and images for: Group Testing for SARS-CoV-2 Allows for Up to 10-Fold Efficiency Increase Across Realistic Scenarios and Testing Strategies
Source: Front Public Health. 2021 Aug 18;9:583377. doi: 10.3389/fpubh.2021.583377 (PMC8416485; doi:10.3389/fpubh.2021.583377)

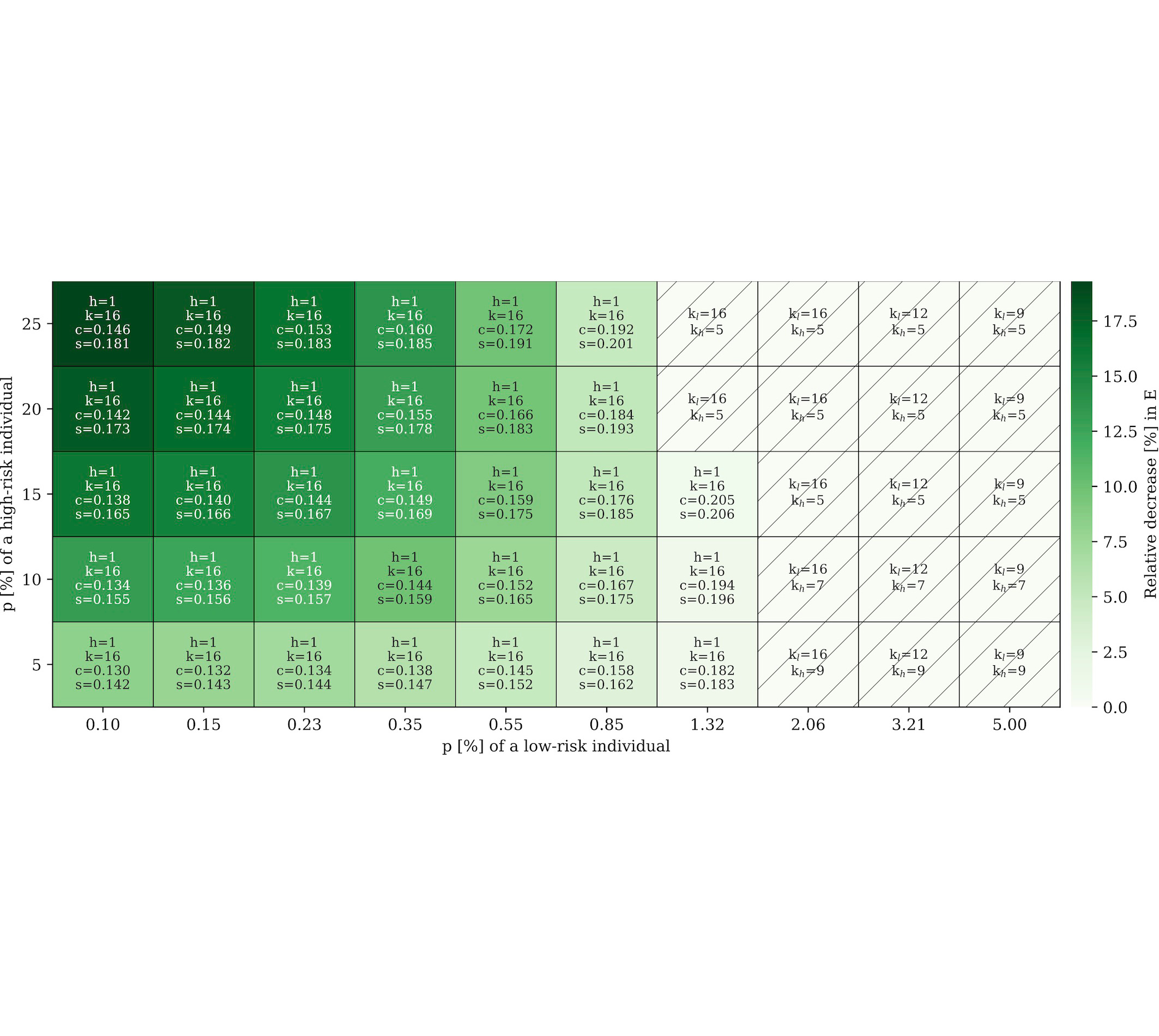

Supplement: Supplementary file 2 [file Image_1.jpg]

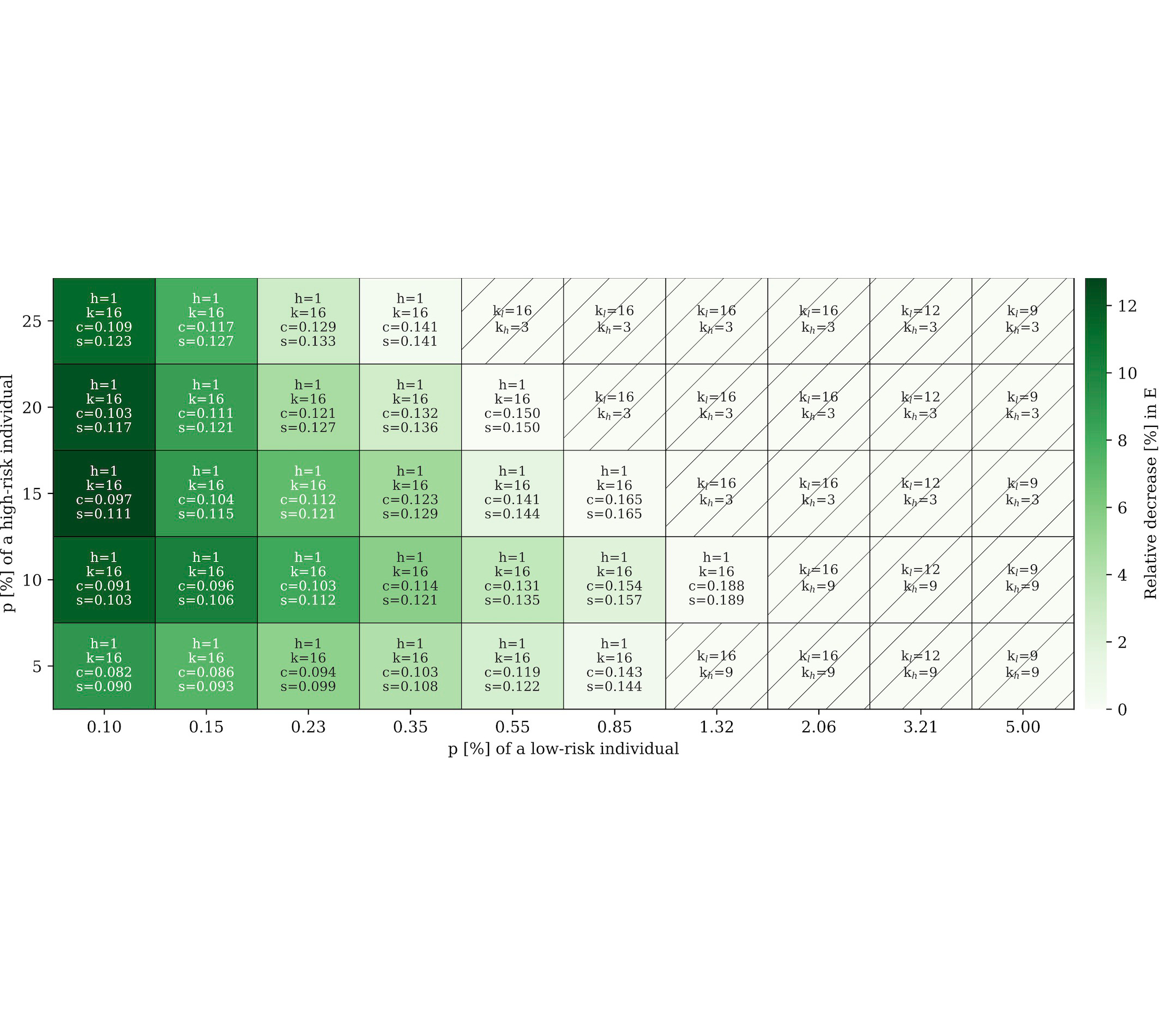

Supplement: Supplementary file 3 [file Image_2.jpg]
